# Human Physiology

**Published:** 1844-05

**Authors:** 


Art. X.—*Human Physiology:* with upwards of three hundred Illustrations. By Robley Dunglison, M.D., Professor of the Institutes of Medicine, etc., in Jefferson Medical College, Philadelphia; attending Physician and Lecturer on Clinical Medicine at the Philadelphia Hospital, &c. &c. "Vastissimi studii primas quasi lineas circumscripsi."—Haller. Fifth Edition, greatly modified and improved. 2 vol. 8vo. pp. 648, 656. Philadelphia: Lea & Blanchard, 1844.

Many will be surprised to see a Fifth Edition of this admirable treatise, so quickly succeeding the fourth. But he who makes books, as well as he who reads them, must be no laggard or sluggard, if he expects to keep pace with science, in these times of stirring and active inquiry. With the discoveries of Bell, physiology and anatomy received an impulse which is still felt; and what with the vivisections of Magendie, the experiments of Brodie, Marshal Hall, Grainger, Reid, &c., the investigations of Ehrenberg, Schwann, Müller, Gerber, Mandl, and others with the microscope, we bid fair to discover some of nature's most hidden and recondite mysteries. Such has been the rapid progress of physiology within a short period, that to make his work a fair reflection of the present state of the science, no less than on account of its extensive popularity, Dr. Dunglison has found it necessary to put forth a new edition, with material modifications and additions.

To those who may be unacquainted with the work, we may say that Dr. D. does not belong to the mechanical, chemical, or vital school exclusively; but that, with a discriminating hand, he culls from each and all, making his treatise something very different from what is indicated by its modest motto—not merely the "first lines," but a very excellent and complete digest of the vast subject.

Of the three hundred illustrations, nearly one hundred have been added to this edition. Besides this, most of the old cuts have been retouched, or replaced by superior ones.

